# How Children and Adolescents Perceive Their Coping With Home Learning in Times of COVID-19: A Mixed Method Approach

**DOI:** 10.3389/fpsyg.2021.733428

**Published:** 2021-11-30

**Authors:** Inga Simm, Ursula Winklhofer, Thorsten Naab, Alexandra N. Langmeyer, Anja Linberg

**Affiliations:** German Youth Institute (DJI), Munich, Germany

**Keywords:** home learning, COVID-19, children and adolescents, student characteristics, coping, mixed-method analysis, family, peers

## Abstract

With the COVID-19 pandemic, children and adolescents confronted a completely new learning situation. Instead of learning in class, they had to cope with home learning to achieve academically. This mixed-method study examines how children and adolescents in Germany perceive their coping success with home learning during the COVID-19 pandemic and how personal, school, family, and peer context factors relate to this self-perceived coping success. Quantitative data from an online survey of *n*=141 children (*m_age_*=10,8y) and *n*=266 adolescents (*m_age_*=15,2y; study 1) were used to analyze the questions with multiple regression analysis. With the qualitative data from 10 interviews with parents and their children (study 2), we examined the process of how school, family, and peer groups interact with students’ way of coping with home learning. Quantitative data show that most children and adolescents perceived their coping with home learning as successful and that school joy before COVID-19, parental support, and available equipment during home learning are still relevant for children, and family climate, calm place to learn, and equipment during home learning are important for adolescents learning at home. Qualitative data show that students apply individual ways of coping with home learning, where family and peers have a vital role, especially when contact with teachers is limited. Quantitative data confirm the importance of family context for students’ self-perceived coping success.

## Introduction

Depending on student characteristics and the context of their support, learning already holds challenges for many students in school (Wang et al., 1993; Doll and Prenzel, 2004; Helmke, 2007). However, with the COVID-19 pandemic, new challenges arose, and many existing ones intensified (Blume et al., 2021). Due to school closures in spring 2020 related to COVID-19 restrictions in Germany, schools no longer offered a learning environment characterized by students’ interactive and analog learning with peers (Schiepe-Tiska et al., 2016a; Eickelmann et al., 2019) under the guidance and support of the teacher (Hattie, 2010). Instead, home learning became the main form of scholastic learning. In Germany, this meant teachers provided exercises, and parents were required to offer learning support, while physical contact with classmates was restricted (Wildemann and Hosenfeld, 2020; Wößmann et al., 2020). Therefore, digital devices gain importance to participate in scholastic learning.

Students, parents, and teachers expected home learning to adversely affect the learning behavior of children and adolescents (Helm et al., 2021). However, initial results showed that students in Germany cope differently with home learning. The amount of time students spent learning was lower than before and showed high variances between students (Wößmann et al., 2020; Helm et al., 2021). At the same time, many students reported having no problems with self-organization (Helm et al., 2021).

A well-studied finding is that students’ states and traits and their school, family, and peer context influence learning outcomes (Wang et al., 1993; Doll and Prenzel, 2004; Helmke, 2007). Like in-class learning, there are more or less supporting variables for children and adolescents’ in-home learning in the COVID-19 pandemic (Müller and Ehmke, 2016). Compared to adolescents, children tend to ask more for support from their parents to cope with a new situation. In line with this finding, parents of children compared to adolescents reported a higher amount of time needed to support students’ home learning in Germany in times of COVID-19 (Wildemann and Hosenfeld, 2020). Further results on learning during the COVID-19 pandemic showed that students who have good grades tend to spend more time learning at home than others. Furthermore, students from higher socio-economic backgrounds report higher perceived learning success, higher learning motivation, more autonomy, and more parental support in home learning. Nevertheless, current studies about home learning (Fickermann and Edelstein, 2020; Huber and Helm, 2020; Thorell et al., 2020) miss out on students’ peer context even though peers play an important role in class learning. Classmates can support each other by motivating or explaining on eye level (Wecker and Fischer, 2014).

Therefore, the present study examines students’ self-evaluation and process of coping successfully with home learning and its relation to students’ support from school, family, and peers. The study applies a mixed-methods design that extends previous research by combining quantitative data about the self-perceived coping success with qualitative data about the process of coping to provide a more complete and nuanced picture than would have been possible with either approach alone. In addition to its value for current research, our paper adds to the knowledge of models of home learning in times of school closures. Finally, considering policymakers, administrators, and school personnel, our paper highlights critical factors that promote or hinder successful home learning.

## Coping with Home Learning

**Figure 1:**
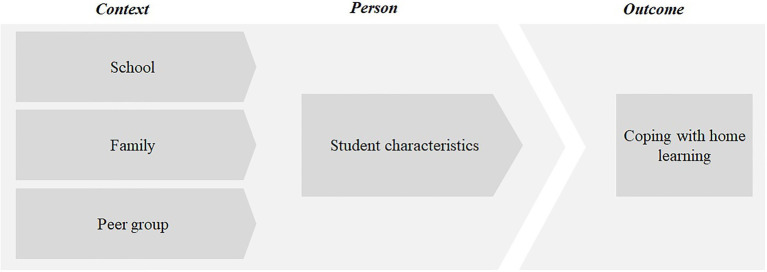
Theoretical model.

Lazarus and Folkman (1984) defined coping as constantly changing cognitive and behavioral efforts to address specific value-related demands. Studies indicate the importance of coping strategies to facilitate positive, adaptive outcomes (Sprang and Silman, 2013). Due to the COVID-19 lockdown, children and adolescents experienced a dramatic shift in how school learning works. To achieve academically in times of COVID-19 lockdown, students must learn to cope with the demands of this new learning situation. Theory and research on coping and learning, therefore, provide a framework to empirically examine students’ coping with learning at home during the COVID-19 pandemic. The way students cope with new situations depend on personal predispositions and their context (La Fuente et al., 2017). Thus, we distinguish in our model between context, person, and outcome (Figure 1).

Like other models of home learning before the COVID-19 pandemic (e.g., Trautwein et al., 2006) and in times of the COVID-19 pandemic (Helm et al., 2021), we consider the impact of student characteristics and school and family context on students’ coping with home learning. Similar to homework, children and adolescents in home learning are confronted with school-related tasks in the home environment. Therefore, homework models (e.g., Trautwein et al., 2006; Moroni et al., 2015) provide a basis for considering the relationships among achievement, homework behavior, homework motivation, student characteristics, parent role, and learning situation. They hypothesize that student characteristics, parent role, and learning situation are central factors in student motivation and behavior, which in turn affect student achievement. The models focus on three main protagonists: students, teachers, and parents. In our theoretical model, we included student peer group as an additional source of help for students in times of home learning.

### Students’ Characteristics: Children’s and Adolescents’ Achievement and Motivational-Affective Prerequisites in the COVID-19 Pandemic

Children and adolescents who have bad grades, do not believe in their own abilities, and do not enjoy learning are more likely challenged with learning (Bandura, 1993; Seidel, 2006; Helmke, 2007). Therefore, students’ academic achievement, self-efficacy, and school joy might also play a central role in successfully coping with home learning. Furthermore, students of different age and gender cope differently with demands.

*Academic achievement* includes students’ success in learning. In school, grades are a conventional way to display students’ academic achievement. Although grades are not without controversy, they appear to be a significant indicator of students’ prior knowledge, engagement, and success in school and a predictor of subsequent professional careers (Elsäßer, 2018). Therefore, it can be expected that students who cope successfully with learning before the COVID-19 pandemic might also cope with home learning in the COVID-19 pandemic.

*Self-efficacy* and *school joy* are essential motivational-affective prerequisites of students. While self-efficacy is one’s subjective belief in the ability to complete specific tasks in school and overcome obstacles and difficulties (Schwarzer and Jerusalem, 1999), school joy is more connected to the affective part of the motivation. Self-efficacy is nurtured by comparing with others and feedback from people in their social background (Bandura, 1977). While self-efficacy and enjoyment are strongly related to students’ academic achievement, believing in one’s abilities and enjoying learning might also help students cope with challenging learning situations. Previous studies showed that children and adolescents who have and believe in having the necessary abilities show more effort and perseverance in learning even when they face obstacles (Bandura, 1993; Parker et al., 2014).

Furthermore, self-efficacious people are more likely to cope with new situations and challenges (Bandura, 1977; Wigfield et al., 2015). Children compared to early adolescents show more self-efficacy, and girls compared to boys tend to underestimate their abilities (Schiepe-Tiska et al., 2016b; Skinner and Saxton, 2019). Moreover, children and girls show a higher tendency to seek the help of others to cope with challenging situations (Skinner and Saxton, 2019). Besides the well-known *gender* differences between boys’ and girls’ self-efficacy, Blume et al. (2021) showed that primary students’ gender also correlates with their task enjoyment in times of COVID-19.

Studies showed variances in students’ academic achievement and motivation during home learning due to the COVID-19 pandemic. A first review showed that between one-fifth and one-half of the young respondents expect negative consequences on their learning success (Helm et al., 2021), while a third of them enjoyed learning at home (Helm et al., 2021). Students who have difficulties organizing and motivating themselves show more problems in coping with learning at home than in school (Becker et al., 2020; Goldan et al., 2020). Parents predominantly describe their children and adolescents as motivated and expected consistent achievements, while most teachers disagree with parents’ impressions (Wildemann and Hosenfeld, 2020; Helm et al., 2021).

The importance of *age differences* is well known for successful coping (Zimmer-Gembeck and Skinner, 2011; Skinner and Saxton, 2019). While children need more help from others (e.g., parents and teachers), more independent adolescents are asked to organize and encourage themselves (Zimmer-Gembeck and Skinner, 2011). In help-seeking, family members are central contact persons in childhood. In the shift to adolescence, peers become more important to ask for help (Berndt, 1999).

From this point of view, we support the need for differentiation between children and adolescents and their achievement and self-efficacy to examine the effect on their coping with home learning.

### Contextual Effects for Children’s and Adolescents’ Coping With Learning in the COVID-19 Pandemic

Besides individual factors, children and adolescents are embedded in contexts. Central factors that describe contexts are relationships with people, processes, and materials (Bronfenbrenner, 1986). Also, being in the same context, these factors can be perceived very differently by different people.

These contexts play a central role in students’ learning (Seidel and Shavelson, 2007; Bronfenbrenner, 2012; Ditton, 2013; Vollet et al., 2017; Skinner and Saxton, 2019; Lyell et al., 2020). The most common contexts that affect children’s and adolescents’ learning are school, family, and peer group.

#### School Context

School is an institutional context that familiarizes children and adolescents with different forms and contents of systematic learning. Schools were ill-prepared for the pandemic-related school closures. They lacked digital infrastructure, especially in primary schools (Eickelmann et al., 2019; Eickelmann and Gerick, 2020).

During this time, teachers offered most students digital learning materials (Huber and Helm, 2020), which they received *via* email, learning platform, cell phone, or video conference (Huebener et al., 2020; Helm et al., 2021). Between 40 and 70% of students report that digital teaching was offered within the first weeks after school closure, while others did not. Furthermore, students perceived teaching as having little cognitive activation or individual support (Helm et al., 2021). Between one-third and one-half of students missed contact with their teachers, stating that personal contact with teachers was rare (Helm et al., 2021).

As the time students spend learning seems to play a central role in school (Seidel and Shavelson, 2007), the variance of time students spend in home learning and contact with their teacher must be considered (Helm et al., 2021). Studies show that video conferences and teacher contact were reported more frequently by students from Gymnasium (Germany’s highest school track) than in other types of schools (Huebener et al., 2020; Helm et al., 2021).

#### Family Context

Besides the school context, students’ family economic, social, and cultural backgrounds have always been directly and indirectly linked to student outcomes and development (Boudon, 1974; Conger and Donnellan, 2007; Helmke, 2007; Bourdieu, 2012). Family stress models and family investment models emphasize the importance of parental economic situation for the development and well-being of children and adolescents (Conger and Donnellan, 2007). This link was often explained by the broader possibilities of sufficient income or certain educational attainment to support children and adolescents (Maaz et al., 2014; Moroni et al., 2015; Müller and Ehmke, 2016) and the stress that economic pressure can cause in families (Conger and Donnellan, 2007). School engagement of parents was likely to enhance students’ school engagement and achievement even for lower performing students (Im et al., 2016). During early adolescence, it is more the indirect forms of parent involvement in school (e.g., parent-child communication about the school, discussing educational aspirations) that are associated with achievement than direct forms (e.g., helping with homework; Fan and Chen, 2001; Hill and Tyson, 2009). Compared to other countries, the direct and indirect relations between students’ socio-economic backgrounds and students’ coping with learning are highly linked in Germany (Müller and Ehmke, 2016).

As the contact with the teacher was limited in times of the COVID-19 pandemic in Germany, many parents reported having helped, especially their children but also adolescents, more in home learning than before (Thorell et al., 2020; Wildemann and Hosenfeld, 2020; Helm et al., 2021). Besides the learning situation itself, the COVID-19 pandemic confronted families with different challenges like financial problems or social isolation that can cause stress. Households with lower socio-economic status and single parents were especially threatened by this (Zinn and Bayer, 2021). Some parents also described their relationship with their children and adolescents as more strained than in the past (Wildemann and Hosenfeld, 2020). In families where the COVID-19 pandemic changed family dynamics and interactions, children and adolescents’ well-being and coping with learning may deteriorate (Achterberg et al., 2021).

Besides the financial burdens families face, parents need to engage with educational media matters: To receive learning material or contact their teacher, students in primary and secondary school need technical equipment. As children and adolescents might not be familiar with handling technical equipment, parents supported particularly young children’s media use (Gerhardts et al., 2020; Züchner and Jäkel, 2021). Most students reported that necessary equipment was available (Wildemann and Hosenfeld, 2020), while between 3 and 25% had difficulties with the availability of technical equipment at home (Helm et al., 2021).

#### Peer Group Context

In recent years, research on learning has included friends, classmates, and peer groups (Kindermann and Skinner, 2009; Wentzel and Ramani, 2016). For peers’ effect on learning, the most common perspective is that peers, like families and school, are sources of motivation, aspiration, and interaction partners (Hanushek et al., 2003). Compared to teachers or parents, peer groups are relationships where children and adolescents can explore, try new things, encourage, inspire, and support each other on eye level (Brandes and Schneider-Andrich, 2017). By functioning as informational support or role models, by motivating each other to hang on to a task, or by stabilizing each other emotionally, peers have been a valuable resource for learning (Youniss, 1994; Hanushek et al., 2003; Kindermann and Skinner, 2009; Ditton, 2013; Brandes and Schneider-Andrich, 2017). Nevertheless, children and adolescents are more strongly influenced by peers with whom they have high-quality interactions (Barry and Wentzel, 2006). It is therefore important that research also focuses on the quality of interactions and how satisfied children and adolescents are with their relationships with their peers.

Due to contact restrictions and social distancing, many children and adolescents did not see their friends and peers physically (Wildemann and Hosenfeld, 2020). Thanks to modern technology, most students reported getting support from friends virtually (Medienpädagogischer Forschungsverbund Südwest, 2020). Previous studies also show that the engagement of students’ peer groups thereby influences students’ school behavior (Kindermann and Skinner, 2009). However, other studies showed that children’s naturally existing peer groups have only a modest effect on academic development (Kindermann and Skinner, 2009).

While based on homework research, central models about home learning in times of the COVID-19 pandemic hardly take students’ peer context into account (Huber and Helm, 2020). Therefore, limited attention has been given to the mechanisms through which peers affect outcomes.

## The Present Study

The current mixed-methods study examines the variance in students’ learning in times of COVID-19 concerning characteristics and contextual resources within their school, family, and peer context. Therefore, this study addresses three research questions:

RQ1: What challenges do students and parents face in home learning, and how do they cope with them?
RQ2: How do individual characteristics (academic achievement, motivational-affective prerequisites, age group, and gender) relate to students’ self-perceived coping success?
RQ3: What effect does school, family, and peer support have on students’ self-perceived coping success?

**Figure 2:**
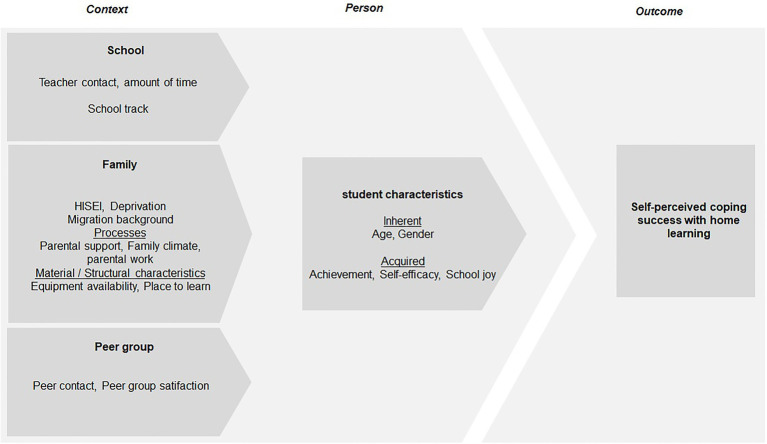
Operationalising model.

Thus far, studies on this topic are limited to either qualitative or quantitative data. With the present study, we use a mixed-methods design to answer our research questions. Mixed-method research collects, analyzes, and mixes quantitative and qualitative data in a single study (Creswell and Plano Clark, 2018). We collected data simultaneously within different sample groups. Study 1 addresses the relationships between individual, family, and institutional contexts with self-perceived coping success (RQ2 and RQ3). For this purpose, we analyzed data from an online survey to validate our theoretical model. Study 2 looks at the individual perspectives of children and their parents on the changes in their (learning) everyday life due to the home learning situation. On the one hand, the study focuses on the personal challenges experienced and how they are overcome (RQ1), but on the other, the study elaborates the importance of parental and peer support for the home learning contexts of the students (RQ3). We addressed these questions through semi-structured interviews with students and their parents. Our interviews focused on students’ learning situations and their strategies to adapt to the new teaching and learning situation and cope with the associated challenges. Especially the role of teachers, parents, and peers can be presented in detail. We integrate the quantitative and qualitative data to gain a better understanding of students’ self-perceived coping success. The operationalization and basis of investigation can be seen in Figure 2.

## Study 1

### Method

#### Sample

Our analysis uses data from the first wave of the large-scale, representative German survey “Growing up in Germany” (Aufwachsen in Deutschland: Alltagswelten; AID:A; Kuger et al., 2021) collected in 2019 and data from a COVID-19-specific add-on module during summer and fall 2020. Both datasets were collected *via* standardized computer-assisted personal interviews. The AID:A 2019 probability sample focuses on 0- to 32-year-old target persons sampled with all household members following a stratified sampling process. For the COVID-19 survey, all target persons were contacted again. Our analysis included all students in general schools (except students with special needs) participating in both waves. This procedure resulted in a total sample size of 407 German children and adolescents from 330 households for study 1, which we analyzed in two subsamples: children aged 9–12years (*n*=141) and adolescents aged 13–18years (*n*=266). Further sample details can be found in Table 1.

**Table 1:** Descriptives.

	Children					Adolescents				
	*n*	*M*	*SD*	Min	Max	*n*	*M*	*SD*	Min	Max
Student’s coping with home learning	141	4.44	1.50	1	6	266	4.47	1.35	1	6
*Students’ characteristics*										
Age	141	10.9	0.96	9	12	266	15.2	1.60	13	18
Gender (0=boy; 1=girl)	141	0.52	0.50	1	2	266	0.53	0.50	1	2
Self-efficacy	140	2.95	0.53	1	4	266	2.89	0.44	1	4
Grade	132	2.09	0.74	1	5	259	2.42	0.78	1	5
School joy	134	3.35	0.71	1	4					
*School context*										
Teacher contact	141	0.34	0.48	0	1	266	0.14	0.35	0	1
Amount of time: home learning	141	2.78	0.63	1	4	265	2.96	0.75	1	4
School track: primary school	141	0.18	0.39	0	1					
School track: gymnasium						263	0.68	0.47	0	1
*Family context*										
HISEI	137	65.1	17.7	22.2	88.7	263	64.4	18.4	11.7	89.0
Deprivation	140	0.23	0.42	0	1	264	0.19	0.39	0	1
Migration background	139	0.20	0.40	0	1	266	0.20	0.40	0	1
Parental support	129	3.51	0.71	1	4	266	2.52	1.05	1	4
Family climate	129	5.21	0.61	3.75	6	265	4.70	0.96	1.50	6
Equipment availability	140	3.71	0.62	1	4	266	3.70	0.58	1	4
Calm place to learn	141	3.51	0.68	1	4	266	3.64	0.64	1	4
Parental work situation	84	0.67	0.47	0	1	120	0.68	0.47	0	1
*Peer context*										
Peer support	113	0.44	0.50	0	1	193	0.78	0.42	0	1
Peer group satisfaction	128	5.41	0.98	2	6	266	5.19	1.08	1	6

#### Measures

Students’ self-perceived coping success, characteristics, school context, family context, and peer group context were assessed by the following variables (for descriptives, see Table 1). Items for students’ characteristics were collected between August and September 2019, while all others were collected in summer 2020. All variables were based on students’ self-report. The COVID-19-specific items focused on the time of strict restrictions at the beginning of the COVID-19 pandemic in Germany, from mid-March to the end of April 2020.

##### Students’ Coping With Home Learning

In order to assess students’ coping with home learning, their perception of its success was assessed *via* a self-developed item. Students reported to what extent the statement “During the time of strict restrictions due to COVID-19, I did well with learning at home” applies to them. Participants indicated their level of agreement on a six-point scale ranging from (1) does not apply at all to (6) does fully apply.

##### Student’s Characteristics

As students’ characteristics, we included inherent characteristics such as *age* in years and students’ *gender* (0=boy, 1=girl) as well as academic achievement and self-efficacy, which were reported in the 2019 survey: students’ mean grade (combined math and literacy grade) in their last report card (in which 1 indicates the best and 6 the worst grade), their *self-efficacy*, which was assessed *via* four items on a four-point scale (e.g., “I can find a solution to any problem”; Cronbach’s alpha_children_=0.57; Cronbach’s alpha_adolescents_=0.68) ranging from (1) is not true – (4) is totally true (Schwarzer and Jerusalem, 1999), as well as in the children’s sample *school joy* (“All in all, I like going to school;” four-point scale (4) does fully apply (1) does not apply at all).

##### School Context

For the school context, we considered student-reported *teacher contact*, which was given if students indicated that during the time of strict COVID-19 restrictions, they had regular contact with their teachers *via* video conferencing or chats (0=no 1=yes). Furthermore, the *amount of time* spent with home learning was considered, for which students were asked how long per day they worked at home for school during the time of strict restrictions (1=not at all, 2=less than 2h daily, 3=2–4h daily, and 4=over 4h daily). In addition, we considered the *school track* in the respective sample. In the children sample, a dummy indicated if children attended primary school in 2019 and in the adolescent sample if the students attended the highest school track (gymnasium; both 0=no; 1=yes).

##### Family Context

As socio-economic characteristics of the family, we included in our analysis *highest socio-economic status in the household* (highest ISEI 08; Ganzeboom, 2010), *migration background* (based on country of birth; 0=neither student nor parent was born abroad, 1=student or at least one parent was born abroad), as well as financial deprivation of the family. Families’ level of *deprivation* was assessed with three indicators. Respondents were asked to indicate whether the following statements applied to their financial situation (1=yes; 2=no because of financial reasons; 3=no because of other reasons): “We can put away money each month,” “We can replace furniture,” and “We can pay for unexpected expenses.” We generated a sum score of all negative replies to these three items (indicating 0=no deprivation; 1=deprivation, counting one or more; Townsend, 1979; Berg et al., 2018). Furthermore, we considered *family climate*, which was assessed *via* four items [e.g., “I like being with my family,” “In our family, we can talk about everything,” on a six-point scale (Cronbach’s alpha _children_=0.76; Cronbach’s alpha_adolescents_=0.58); Moos and Moos, 1981]. Moreover, students reported for the time of strict COVID-19 restrictions, the frequency of *parental support* of their learning at home, if all needed technical *equipment were available*, and if they had a *calm place to learn* (1=never, 2=sometimes, 3=often, and 4=always). Furthermore, the *parental work* in times of COVID-19 pandemic (0=no; 1=yes) was reported using parents’ information if they use any possibility to work less (e.g., short-time work and vacation).

##### Peer Context

For peer context, we included two single items. Students provided information about their *peer group satisfaction* by answering how satisfied they currently are with their peer group. They could indicate this on a six-point scale ranging from (1) not at all satisfied to (6) totally satisfied. In addition, they were asked in a multiple response question whom they had asked for advice and support in difficult situations during the period of severe restrictions imposed by COVID-19. We coded *peer support* (0=no; 1=yes).

#### Analytical Strategy

A stepwise and robust multigroup regression analysis was run to examine the variance of students’ self-perceived coping success with home learning and its relation to students’ characteristics and context. Covariances and clustered SEs were used to take the nested data structure (children in households) into account. Missing values of the variables (max. 27% for peers support) were treated using full information maximum likelihood estimation (Enders, 2001; Acock, 2013) and all valid information of all observations with Stata 15. Analyses were performed separately for primary and secondary school children.

### Results

#### Descriptive Results on Children’s and Adolescents’ Self-Perceived Coping Success

Descriptive results demonstrate that, on average, children (*M*=4.44; *SD*=1.50) and adolescents (*M*=4.47; *SD*=1.34) assessed their coping with home learning to be good. However, 22.0% of children and 21.8% of adolescents reported low values (scale level 1–3) in self-perceived coping success with home learning. Within their home learning, children received exercises from their school digitally (90.8%) and in person or by mail (34.0%). Almost all adolescents reported having received exercises from their school digitally (99.6%), and 13.9% of adolescents received exercises in person or by mail. Furthermore, 34.8% of the children and more than half of adolescents were frequently in contact with their teachers by video or chat.

To examine the role of family context for students’ self-perceived coping success with learning in times of COVID-19 (Q3), correlations within children’s (see Table 1, Appendix) and adolescents’ (see Table 2, Appendix) family contexts are reported. There is a significant correlation between children’s and adolescents’ equipment availability with HISEI (*r_children_*=0.27, *p*<0.05, and *r_adolescents_*=0.16, *p*<0.05) and deprivation (*r_children_*=0.35, *p*<0.001, and *r_adolescents_*=−0.32, *p*<0.001). For children, there is a strong correlation between equipment availability and parental support (*r_children_*=0.24, *p*<0.01) and between calm place to learn and deprivation (*r_children_*=−0.28, *p*<0.001). For adolescents, the highest correlation is between calm place to learn and family climate (*r_adolescents_*=0.35, *p*<0.001). Further bivariate correlations are displayed in Table 1 for children and Table 2 for adolescents.

#### The Relation Between Children’s and Adolescents’ Self-Perceived Coping Success With Their Characteristics and Context: Multiple Regression Analysis

A multiple regression analysis was applied regarding children’s and adolescents’ self-perceived coping success with learning and the effect of individual characteristics and school, family, and peer support (RQ2 and RQ3). Table 2 summarizes its results by reporting standardized coefficients (*β*), SE, and *p* values (*p*) for children and Table 3 for adolescents.

**Table 2:** Individual and contextual predictors of children’s self-perceived coping success with home learning.

Predictors of student’s coping with home learning	Model 1			Model 2			Model 3			Model 4			Model 5		
	*β*	*SE*	*p*	*β*	*SE*	*p*	*β*	*SE*	*p*	*β*	*SE*	*p*	*β*	*SE*	*p*
*Students’ characteristic*															
Age	−0.024	0.089	0.786										−0.059	0.101	0.561
Gender	−0.039	0.082	0.636										0.016	0.083	0.851
Self-efficacy	0.090	0.087	0.303										0.110	0.090	0.219
Grade	−0.151	0.115	0.192										−0.029	0.096	0.764
School joy	0.160	0.081	0.050										**0.200**	**0.080**	**0.014**
*School context*															
Teacher contact				0.071	0.083	0.387							0.108	0.080	0.179
Amount of time: home learning				−0.030	0.076	0.690							0.029	0.077	0.708
School track: primary school				−0.041	0.083	0.623							−0.004	0.100	0.968
School track: gymnasium															
*Family context*															
HISEI							0.092	0.077	0.233				0.040	0.085	0.638
Deprivation							0.061	0.095	0.519				0.033	0.100	0.744
Migration background							0.046	0.106	0.663				0.013	0.129	0.920
Parental support							0.133	0.081	0.103				**0.168**	**0.082**	**0.042**
Family climate							0.044	0.081	0.589				−0.080	0.090	0.372
Equipment availability							**0.238**	**0.116**	**0.040**				0.204	0.114	0.074
Calm place to learn							**0.201**	**0.102**	**0.049**				**0.220**	**0.104**	**0.035**
Parental work							−0.095	0.098	0.331				−0.130	0.106	0.223
*Peer context*															
Peer support										−0.067	0.089	0.454	−0.052	0.093	0.579
Peer group satisfaction										0.155	0.094	0.099	0.121	0.103	0.242
***R*** ^***2***^	**0.075**			**0.007**			**0.185**			**0.028**			**0.294**		

*n*=141 children. *β*=standardized coefficients, *SE*=robust standard error, bold=*p*<0.05; and gender (0=boy; 1=girl).

**Table 3:** Individual and contextual predictors of adolescents’ self-perceived coping success with home learning.

Predictors of student’s coping with home learning	Model 1			Model 2			Model 3			Model 4			Model 5		
	*β*	*SE*	*p*	*β*	*SE*	*p*	*β*	*SE*	*p*	*β*	*SE*	*p*	*β*	*SE*	*p*
*Students’ characteristics*															
Age	−0.098	0.056	0.080										−0.090	0.050	0.073
Gender	0.077	0.060	0.197										0.073	0.059	0.214
Self-efficacy	0.094	0.060	0.120										0.033	0.066	0.618
Grade	**−0.278**	**0.056**	**0.000**										**−0.228**	**0.054**	**0.000**
School joy															
*School context*															
Teacher contact				−0.001	0.066	0.926							0.011	0.059	0.847
Amount of time: home learning				**0.148**	**0.072**	**0.039**							0.030	0.063	0.631
School track: primary school															
School track: gymnasium				0.036	0.064	0.575							−0.048	0.061	0.430
*Family context*															
HISEI							0.000	0.062	0.997				−0.033	0.061	0.589
Deprivation							−0.004	0.074	0.958				−0.017	0.075	0.809
Migration background							0.004	0.060	0.946				−0.036	0.075	0.630
Parental support							−0.018	0.059	0.761				−0.074	0.058	0.201
Family climate							**0.216**	**0.066**	**0.001**				**0.160**	**0.071**	**0.024**
Equipment availability							**0.156**	**0.061**	**0.010**				**0.159**	**0.060**	**0.008**
Calm place to learn							**0.225**	**0.072**	**0.002**				**0.211**	**0.073**	**0.004**
Parental work							0.051	0.091	0.057				0.050	0.097	0.603
*Peer context*															
Peer support										0.043	0.082	0.603	0.054	0.080	0.498
Peer group satisfaction										**0.183**	**0.080**	**0.022**	0.078	0.076	0.303
***R*** ^***2***^	**0.105**			**0.024**			**0.174**			**0.040**			**0.246**		

*n*=266 adolescents. *β*=standardized coefficients, *SE*=robust standard error, bold=*p*<0.05; gender (0=boy; 1=girl).

In Model 1, children’s characteristics explained 8% of the variance in their self-perceived coping success with home learning (*R^2^*=0.08). For adolescents, their characteristics explained 11% of the variance in their learning success (*R^2^*=0.11). Adolescents’ grades negatively predicted their self-perceived coping success with home learning (*β*=−0.28, *p*<0.001) as grade 1 in the German school system is “very good,” and six is “insufficient.”

Regarding students’ and adolescents’ school context only (Model 2), between 1% for children’s (*R^2^*=0.01) and 2% of adolescents’ (*R^2^*=0.02) variance in their self-perceived coping success with home learning can be explained. For adolescents, the amount of time they spend in home learning positively predicts their self-perceived coping success with home learning (*β*=0.15, *p*<0.05).

With variables of students’ family context (Model 3), 19% of the variance for children’s self-perceived coping success with home learning (*R^2^*=0.19) and 18% of adolescents’ self-perceived coping success with home learning (*R^2^*=0.18) were explained. Adolescents’ self-perceived coping success with home learning is predicted by their family climate (*β*=0.22, *p*<0.01), equipment availability (*β*=0.16, *p*<0.05), and calm place to learn (*β*=0.23, *p*<0.01).

In Model 4, children’s and adolescents’ peer groups did not explain any variance in their self-perceived coping success with home learning (*R^2^*=0.00). Adolescents’ self-perceived coping success with home learning is predicted by peer group satisfaction (*β*=0.18, *p*<0.05).

Parental support (*β*=0.18, *p*<0.05) and equipment availability (*β*=0.26, *p*<0.05) positively predict children’s self-perceived coping success with home learning.

In the overall Model 5, which contains students’ characteristics, school context, family context, and peer context, between 29% of children’s (*R^2^*=0.29) and 25% of adolescents’ (*R^2^*=0.25) variance in their self-perceived coping success with home learning can be explained. For children’s self-perceived coping success with home learning, parental support (*β*=0.18, *p*<0.05) and calm place to learn (*β*=0.21, *p*<0.05) are significant. For adolescents’ self-perceived coping success with home learning, their grade (*β*=−0.23, *p*<0.001), family climate (*β*=0.16, *p*<0.05), equipment availability (*β*=0.16, *p*<0.01), and calm place to learn (*β*=0.21, *p*<0.01) are significantly predictive.

## Study 2

The second study sheds light on the subjective experiences of families during school closures due to the COVID-19 pandemic. The focus is on reconstructing the strategies children and their parents developed to adapt to the new teaching and learning situation and cope with the associated challenges.

### Method

Semi-standardized telephone interviews with children and their parents provide the empirical basis for this study. The interviews were conducted in May and June 2020 as part of the study by Langmeyer et al. (2020) on the living situation during the first COVID-19 lockdown in Germany. Participating families were recruited *via* a corresponding supplemental question at the end of the quantitative survey (cp. study 1). We selected a quota sample of 21 families from the group of 2,798 parents who agreed to participate in a supplemental qualitative interview of parents and children as part of study 1. Quota sampling was based on the gender of the children (50% girls), the degree of urbanization of the place of residence (two-thirds urban, one-third rural), siblings (two-thirds with, one-third without siblings), and the age of the children (between 6 and 14years). In addition, we included families’ socio-economic background (perceived coping with income) and state of residence in Germany in the sampling procedure. We included 10 interviews with children between the ages of 10 and 14, including four girls and six boys, in the analyses for this paper. The parent interviews were conducted with the mother of the participating child in nine families, and in one family, the corresponding interview was conducted with the father. Eight of the 10 families have more than one child. About the care situation, the families implemented different models. In four families, one parent is mainly responsible for the care tasks (three mothers, one father); in five families, both parents share the childcare, and in one family, the child is cared for by family friends. Table 4 provides an overview of the sample.

**Table 4:** Composition of the qualitative sample.

Aliases	Age	Gender	Grade	Siblings	Parental interviewee	Educational background	Parental care situation
Maja	11	Female	5th grade	Brother (18y)	Mother	University degree	Mother responsible for care work; works 10h per week as cleaner father: gastronomy management
Benny	11	Male	5th grade	Two brothers (6y, 9y)	Father	University degree	care work is shared between father and mother
Heike	11	Female	5th grade	No siblings	Mother	University degree	mother works full time in home office; father, who lives separately, takes over a substantial part of the care tasks
Jan	14	Male	8th grade	Sister (11y)	Mother	University degree	mother is primarily responsible for childcare; father works in home office
Marcus	10	Male	5th grade	No siblings	Mother	Master craftsman certificate	both parents work full time; Marcus is cared for by family friends
Lars	11	Male	5th grade	Two sisters (8y, 14y)	Mother	University degree	care work is shared between father and mother
Andrea	11	Female	6th grade	Sister (9y)	Mother	University degree	both parents work full time in homeoffice
Maria	11	Female	6th grade	Brother (8y)	Mother	University degree	care work is shared between father and mother
Jonas	11	Male	5th grade	Three adult siblings; brother (24y) living in the family household	Mother	University degree	care work is shared between father and mother; older brother supports parents‘care work
Thomas	14	Male	8th grade	Brother (12y)	Mother	University degree	mother is primarily responsible for childcare; father works one day per week in homeoffice

Considering the interview procedure, the interviews with the parents and the interviews with the children started with an open-ended question that addressed current changes in their everyday life due to the COVID-19 pandemic. This approach provided a first impression of the most important aspects of the issue from an individual perspective and the general mood in the family. After that, the interviews focused on the circumstances of homeschooling, the possible restart of school, and the family’s general childcare situation. Furthermore, children and parents provided information about the living situation, siblings, grandparents, and friendships, and activities and mood in the family. The interviews took about 40–60min per family, where the interviews with the children lasted between 20 and 25min. All participants found the interviews a welcome opportunity to talk about their experiences during the COVID-19 pandemic. Although studies with children present particular challenges for researchers (Ólafsson et al., 2013; Wagner, 2016), the older children included in our analysis were remarkably detailed and open about the changes in various aspects of their daily lives and experiences related to the crisis.

### Analytical Strategy

The texts were transcribed and completely anonymized. The analysis followed the procedure of content structuring qualitative content analysis (Kuckartz, 2018). The categories for the analysis were formed both deductively from the questionnaire and inductively from the interview material. Coding and analysis of the interviews were done by three researchers using MAXQDA software. Tables 3, 4 in the Appendix of the paper present the final categories, their descriptions, and anchor examples from the interviews with the children and parents, respectively.

### Results

The analysis of the interviews allows for a more detailed insight into how children and parents experience the situation of home learning. Despite the abundance of material gathered from conversations with the children and their parents, at this point, complementary to the findings of study 1, three central arguments will be elaborated. Considering RQ1, we elaborate children’s perception of home learning challenges they faced at the onset of the COVID-19 pandemic. Subsequently, we elaborate parents’ perspectives on their performance in ensuring their children’s participation in school-based educational opportunities and their view of school support services for their children during home learning (RQ3). Finally, we shed light on the importance of peers and their contribution to students’ coping with the home learning situation (RQ3).

#### Children’s Perceptions of Home Learning Challenges

In the interviews with children, it is clear that pandemic-related home learning represents a profound change in their daily lives. This is particularly evident in the detailed narratives about the challenges posed by the changed living situation. On the one hand, the children’s reports draw comparisons to the pre-COVID situation, and on the other, they refer to newly developed routines and ways of successfully coping with the situation.

The loss of school attendance due to contact restrictions unsettled many children at the beginning of the first lockdown (Table 4, cat. 1a). Benny (11y) experienced the whole situation as being “very uneasy and critical”…“because you did not know exactly what to do now.” Physically attending school as a place of learning has been an essential part of the children’s daily routine, while the home has been a place of preparation and follow-up for school. Therefore, a key challenge initially was to coordinate the conditions and processes for successful home learning between children, parents, and school. Thus, it is not surprising that the children frequently bring up that they can learn better at school. They miss their teachers’ explanations that increase their understanding of learning materials. In addition, the school setting helps them concentrate and motivates them better. In this context, students feel overwhelmed by many tasks with mostly little support from the school. “We get the assignments from school by email, and you have a bit of a feeling that you somehow get more assignments than you would normally if you went to school, and you also cannot learn or understand things as well as in normal lessons” (Maja, 11y).

Considering the rearrangement of their daily routines, the students developed a new daily structure with the support of their parents, who set working hours and partly helped them maintain work discipline (Table 4, cat. 4a; Table 3, cat. 4a). Exemplarily, Benny (11y) describes the difficulty of the challenge to self-organize and complete a multitude of tasks independently: “How am I supposed to manage all the tasks? That was a big problem at the beginning, but then I and my mother also found appropriate solutions with apps that show the time, that I worked for 3h and 30min every day. And then we also made plans, and then also with such a list to check off, which then worked very well with school after a while.”

Against this background, it is not surprising that the children report difficulties concentrating and maintaining motivation (Table 4, cat. 1b). While it is a drastic experience to be left alone with the challenge of successfully completing a multitude of school tasks, distractions from mobile phones, television, and other media are within reach. Consequently, some students fear the risk of missing a large amount of learning. Maria (11y) reflects this situation as “When I am home alone […] it is difficult to concentrate […]. It is the same at school, but at home, I have something like a mobile phone or TV, where I would rather do that and think it does not matter so much if I miss 1day, but there [at home] I somehow miss 3days.”

In contrast, it is helpful if lessons are held *via* video conference (Table 4, cat. 3), which was usually only offered very sporadically in this first phase of school closures, which the students regret: “I would have actually wished for a bit more, because I always thought it was such a change, not just sitting at a desk, because I somehow could not motivate myself so well to just do homework alone at a desk, so I thought that was pretty cool” (Jan, 14y).

Despite challenging conditions, the students also describe experiences of coping successfully with learning (Table 4, cat. 2). Marcus, who worked with his friend on their school tasks, proudly reported, “We managed everything that was assigned, even additional tasks.” In addition, children with special needs can benefit from the intensive support of their parents – as long as they have time for it. Heike, who has a learning disability, reported that with intensive support from her father, “I learned a bit more because I could ask more questions.”

#### Support From Parents – Parents’ Perspective

Parents face the challenge of finding new arrangements in balancing work and family life. In addition to possible pandemic-related changes in their daily work lives, school closures have put parents on notice to support their children’s home learning. Against the background of our third research question, we asked parents their perspective of the support needs and processes for their children’s home learning (RQ3). Our interviews with parents revealed that accompanying the children in home learning was very demanding and time-consuming (Table 3, cat. 1a), especially, the coordinating of professional and childcare while working from home led to stressful situations, as Benny’s father describes: “You cannot look after three children and work 7h at the same time, it does not work like that. […] Yes, of course, it is a burden, the food has to be cooked, both children have to be taught, the third one also wants his attention and to be looked after. So that is already a higher burden than normal weeks” (Father of Bernd, Jonas, and Benny, 6y, 9y, and 11y).

From the parents’ point of view, the extent of support needed in home learning depends mainly on their children’s ability to solve home learning tasks independently (Table 3, cat. 2) and the amount of support from the school (Table 3, cat. 3). Considering the ability to learn independently, parents often contrasted older and younger siblings. While younger children of primary school age (6–10y) needed intensive guidance with explanations and substantial instructions to solve given tasks, pupils in secondary school were sometimes already able to work quite independently. In this context, the interviews show that parents had to support their children in dealing with digital technology and digital work processes (Table 3, cat. 4c; Table 4, cat. 4c). Bernd’s father reports, “My wife, in particular, structured [incoming tasks; …] and then printed everything out accordingly and put it down and then sent it back again if necessary” (Father of Bernd, Jonas and Benny, 6, 9, and 11y). The mother of Jan (14y) confirms that her daughter, in particular, has “no connection to it at all yet.” In addition to a tight time budget, the families interviewed hardly have the spatial and technical infrastructure to enable all family members to use a digital workplace undisturbed. At least, as Andrea’s mother summarizes the technical challenges, Andrea (11y) “has also learned a lot about media and IT, so that she can print or scan what she needs herself and send it back. […] [B]ut for an elementary school child, [Andrea’s sister (9y)], that means […] [however] being asked over and over again, ‘What do I do now?’ and why something did not work […]. And that alongside somehow teleconferencing and email […] and that really makes you very tired.”

Directly related to their children’s independence in home learning, parents see the level of support their children receive from the school (Table 3, cat. 3). The more interactive the exchange with the school and the more frequently parents perceive feedback from teachers, the less intensively they report their excessive demands in our interviews. Two cases can be contrasted here; on the one hand, Andrea’s mother, who reports that the school was able to switch to digital teaching very quickly: “In Andrea’s class, it works very well, almost from day one they switched to online school. They are well looked after, and accordingly, we have to do less,” on the other, Jan’s (14y) mother compares the situation of her two children. Concerning the younger daughter (11y), she describes that the lack of contact with teachers resulted in a high need for support: “Well, we have experienced teachers where you really have the impression that a bunch of worksheets is emailed without any instructions, explanations, help, and also without demanding feedback, and you had the impression, well, the child is now working for the thick folder, so to speak, and does not know at all what for. And so I then partly did not succeed at all in motivating her, only with lots of sweets and any promises.”

Based on this statement, which many of the interviewed parents shared, it becomes clear that supporting the children was also about maintaining motivation, concentration, and work discipline (Table 3, cat. 4b; Table 4, cat. 4b). Maja’s mother, for example, reported, “The first few times [of home learning], I sat with her and did some of the tasks with her or just watched her so that she did not distract herself.”

One aspect parents and children rarely address in the interviews is content support for their children (Table 3, cat. 4d; Table 4, cat. 4d). Due to their comparatively high level of education, parents are unlikely to have any problems following their children’s schoolwork content. Instead, the lack of time to deal with home learning is a more significant issue (Table 3, cat. 1b). Especially families with unique burdens reach their limits under the conditions of school closures. It is their children who suffer from a lack of support. Exemplary is the case of Maria, whose parents are at capacity by accompanying her younger brother, who has special needs due to ADHD: “I think that if we were to sit next to her as intensively as with her brother […], she would certainly be able to do more […], but that is simply not possible. […] We simply had to set priorities so that we could keep Hannes (8y) on track somehow, who has problems at school anyway. And unfortunately, that is at the expense of Maria’s school; it has to be said quite clearly. But on the other hand, we have to get our work done.”

#### Support From Peers

Through the narratives of the children and adolescents, it becomes clear that connectedness and support from peers is a central strategy to cope with the new demands of home learning (RQ3). Networking with classmates and friends makes it easier to cope with school tasks. At the same time, interacting with peers has a positive effect on motivation and mood. Using media plays a crucial role in maintaining contact, be it telephoning, writing text messages, or networking *via* online platforms (Table 4, cat. 5a). For example, Jan (14y) reports: “Yes, we have such a program, so ‘Discord’ is the name of it, which we also always use when we play computer games or something (laughs), and then we always met at a time and just did the tasks together.”

Considering home learning challenges, students seek mutual support with questions about understanding the assignments, helping with explanations, or giving tips when solving difficult tasks (Table 4, cat. 5c). Marcus (11y) describes how mutual support leads to success when working together with his friend: “So, for example, if my friend did not know something or I did not know something, then we gave each other tips or something about what it could be, and then we sometimes, so often, came up with the right solution.” For Marcus, who comes from a non-academic home, an additional strategy is to ask the “smartest” students from the class for advice: “We also wrote a lot like that, if tasks were not clear so that I and my friend did not know and his mother did not know either, we sometimes asked the smartest from our class.”

Maja (11y) admits, “We talked a lot on the phone […] and then just did the tasks together when studying because it is just a bit stupid alone” (Maja, 11y; Table 4, cat. 5b). Thus, the class is strengthened as a social community if the digital platforms of the schools enable pupils to network with each other. Overall, it becomes apparent in the interviews that support from the peers and the contact associated with it contributes to increasing the motivation for home learning and conveys the feeling of self-efficacy in successfully and independently coping with this situation.

## Discussion and Conclusion

At first glance, the COVID-19 pandemic has significantly changed the way children are schooled. In Germany, face-to-face instruction has been replaced by various formats of home learning, in which parents primarily support their children in coping with school obligations. With this in mind, this paper argues that the home learning situation can be described as analogous to parental monitoring of their children’s homework. In the literature review, we identified factors that describe the extent to which children cope with the home learning situation. We illuminate this general thought through the presentation of two studies in a mixed-methods approach that examines how students cope with the home learning situation during school closures due to the COVID-19 pandemic in Germany. Study 1 shows, through a quantitative online study, that children with differences in school joy, parental support, and quiet place to study, and adolescents with differences in grade, family climate, availability of technological devices, and a quiet place to study cope differently with home learning. The results of the second study highlight, using qualitative telephone interviews with children and youth, students’ challenges in solving and completing their assignments, time management, work discipline, and self-motivation. It also reveals that children and adolescents ask parents and peers for help. Parents experienced answering their children’s calls for support as an immense effort. In line with previous findings of home learning in times of the COVID-19 pandemic (Huber and Helm, 2020; Wößmann et al., 2020), children’s and adolescents’ characteristics and their school, family, and peer context affect how students cope with the new home learning situation. Whereas in study 1, based on the mean values, it seems that children and adolescents are coping quite well with the learning situation at home, Study 2 shows that the changed learning situation is accompanied by a series of adaptations, which are also clearly stressful for students.

Nevertheless, we discuss what factors are essential for home learning due to the COVID-19 pandemic and whether models on homework and learning in class are appropriate. Based on research on homework (Trautwein et al., 2006; Moroni et al., 2015), we choose a model that incorporates student characteristics and student contexts. Among the students’ characteristics, the pre-pandemic grade was found to be particularly significant for adolescents. It is noteworthy, however, that this effect is absent for children. In addition, our results show that children’s enjoyment of school before the COVID-19 pandemic contributed to their ability to cope better in the current learning situation at home. Apparently, children and young people who were already better at learning before the COVID-19 pandemic are finding it easier to do so during the pandemic. This result fits with the findings of the interview study that besides solving tasks and completing them, students need to develop new structures in their time organization and a new form of work discipline, for which, in turn, concentration and motivation are essential prerequisites.

In contrast to previous research on homework, which emphasizes the importance of teachers and exercises (Trautwein et al., 2006), in study 1, it seems that the school context is less important. In study 2, it becomes clear that the exchange between teachers and students is central to how motivated the students are. The new, unfamiliar situation is particularly stressful because the students have to work alone for the school. In this respect, coming to terms with this situation is also about overcoming the isolation of being “alone at your desk” and creating social connectedness. One possible explanation why the school context is less important in study 1 than reported in previous studies on homework is that home learning has radically changed in times of the COVID-19 pandemic, and the previous results are not directly transferable. Another explanation could be that we only looked at the first period of home learning with the study: Possible differences between different school contexts, such as differences in the type of school, were not (yet) visible. In this first, early phase of the pandemic in Germany, many parents worked at home or were on reduced hours and thus may have been able to support their children in their learning. However, the differences may also be due to our operationalization of the school context with few variables. Furthermore, the study mainly analyzes the quantity of contact and the working time. The quality, on the other hand, was not part of the quantitative study. However, this could also be decisive for the children and adolescents’ perceived coping ability.

Concerning the family context, in study 1, the most important factor is equipment availability and a calm place to learn. If the children and young people have the essential work resources for home learning, such as computers or laptops, printers, and a calm place, the self-perceived coping success with home learning is greater. The bivariate correlations show that children in deprivation are less well equipped than other children and deprived children report lower coping success. The regression analysis suggests a mediation effect, as the effect of deprivation is removed when equipment availability is controlled for. Thus, it can be stated that children in deprivation are disadvantaged by not having all the necessary resources at their disposal, which affects their self-perceived coping success with home learning. Future research needs to observe whether these results in more substantial long-term disadvantages than children in deprivation already have.

Interestingly, we observe a link of the family climate to coping success only for adolescents. If the adolescents experience the climate in their families as positive with strong cohesion, they also seem better prepared for the new challenges. For children, this connection seems to have less significance, which might be due to the fact that most of the children consider their family climate to be particularly positive, while adolescents are more critical of their family climate.

Regarding the importance of children’s and adolescents’ support in their family and peer context, we found a discrepancy between qualitative and quantitative data. While in study 1, we see less importance of students’ family and peer context for students’ self-perceived coping success with home learning, it becomes clear in study 2 that this context does have significance in the subjective reconstructions of the situation. Thus, the interview study shows that this means not just explaining the tasks correctly. Parents are challenged in their competencies regarding the current learning material and as “managers” of home learning by helping the children organize the working day, divide and structure the school tasks, and keep track of the work tasks that have been done and those that still need to be done. Furthermore, they often have to act as “motivation coaches” and support concentration and motivation. An explanation for the discrepancy between the quantitative and qualitative data might be that parents can provide support without their children and adolescents noticing it. Suppose parents can support adequately; this changed learning situation can also have positive aspects: At least one of the interviewed children (Heike) reports advantages of learning at home. She is better supported there than at school due to the 1:1 learning situation.

Considering the significance of peers in home learning situations, research literature in the context of the COVID-19 pandemic appears sparse. While study 1 suggests, at least in the overall models, that satisfaction with the peer group and peer support are not significant contributors for the young people’s coping with home learning, study 2 clarifies that networking and cooperation with peers is an essential strategy to cope with the new situation and new demands. In line with previous research, support from friends and classmates not only helps in a very concrete way to understand and solve tasks but also contributes significantly to overcoming the feeling of being alone and isolated and to creating social connectedness (Lyell et al., 2020; Sun et al., 2020). Furthermore, the interviews indicate that support from classmates is essential for children from families without an academic background, where parents cannot provide support in terms of explanations of the learning material. This shows that the role of peers is not negligible and should be looked at more deeply, especially in quantitative studies of learning at home, which have been rather neglected so far.

Comparing the three contexts in our studies, it seems that the family context is very important for self-perceived coping success with home learning. This result is an all-important finding, as it is well known that family background has always played a significant role in school success in Germany (Müller and Ehmke, 2016). Thus, the COVID-19 pandemic is likely to exacerbate existing disadvantages among children and youth. It can be assumed that similar results will be seen in Germany as in the Netherlands, where it could be shown that the pandemic results in losses in learning success, especially for children from disadvantaged backgrounds (Engzell et al., 2021). Therefore, it is important that special attention is paid to these children after the COVID-19 pandemic and that they are given unique opportunities to catch up on their learning.

Considering the theoretical framework, our studies suggest that homework theories (Trautwein et al., 2006; Dumont, 2012) only partially represent the COVID-19 situation. Student characteristics, school characteristics, and the role of parents are indeed important in the current COVID-19 period. However, the respective characteristics are partly different than in the homework situation. This is hardly surprising, as the home learning situation is different from the homework situation: Students spend more time alone doing exercises without getting much feedback than in the pre-pandemic homework situation. Furthermore, the digital changes of the school situation can influence students’ achievement and motivation in home learning. Mainly, younger pupils must first acquire the corresponding skills and competencies. In line with the homework model by Trautwein et al. (2006) or the home learning model of Huber and Helm (2020), parents play a central role. Nevertheless, compared to checking children’s and adolescents’ tasks, it might be more challenging to support and accompany children in this often new digital learning space. Especially in study 2, the importance of peers becomes visible; this has rarely been considered in the homework literature so far but does not appear in recent models developed for home learning (Huber and Helm, 2020). It is important that this aspect is taken up in both theoretical considerations and quantitative studies.

## Limitations

Even though the present study provides added value to the existing literature, the study’s limitations should also be noted. First, the conclusions of the present analysis are restricted to the study’s sample. Second, it should be noted that the AID:A COVID-19-specific add-on was only able to reach a portion of the respondents in 2019. Third, students from the highest school track are overrepresented among our sample. We took into account students up to the age of 18, and students in Germany’s highest school track go to school for a longer period of time than in other German school tracks. Fourth, our study is only cross-sectional, limited to the period of the initial lockdown. As a result, no causalities can be verified. Longitudinal data are also needed to examine the interaction of the different contexts. The first study shows that school and family context differs between the first time of strict school closures in Germany due to the COVID-19 pandemic and the second time of school closures (Wößmann et al., 2021). Therefore, to get a more holistic view of students’ learning situation due to the COVID-19 pandemic, the different periods should be regarded.

Furthermore, the construct of *self-perceived coping success with home learning* was only measured with one single indicator. Additional information about students’ learning success in the form of grades or tests would be a valuable source for getting more insights into the long-term effects of students’ coping with home learning and their performance. The problem of single indicator measures also applies to some predictors in our models (e.g., peer support). This could also be an explanation for the fact that some correlations do not occur as expected. Finally, it should also be noted that no extensive information was available on the individual contexts, so possible central aspects were not taken into account here, which is why the school context, for example, appears to be of little importance.

## Outlook

Nevertheless, the study offers starting points for further research as well as for practical work with children. As already mentioned, the disadvantages of certain groups of students have to be made up for in the post-pandemic period. If the pandemic lasts longer, or if other situations arise in which learning at home is necessary, it must be ensured that the family is not ascribed such a central role in the learning situation, in addition to adequate equipment necessary for all students. Not all families can adequately support their children. It was already true before COVID-19 and is becoming even more critical in times of home learning.

## Data Availability Statement

Publicly available datasets were analyzed in this study. This data can be found at: doi: 10.17621/aida2009.

## Ethics Statement

Ethical review and approval was not required for the study on human participants in accordance with the local legislation and institutional requirements. Written informed consent to participate in this study was provided by the participants’ legal guardian/next of kin.

## Author Contributions

IS, TN, ALa, and ALi planned study 1. UW planned study 2. All authors wrote the manuscript. IS and ALi prepared the data and performed all statistical analyses regarding study 1. Considering study 2, UW and TN prepared the data and performed all analyses. All authors contributed to the article and approved the submitted version.

## Funding

Data collection has been supported by the German Youth Institute, which is funded by the German Federal Ministry for Family Affairs, Senior Citizens, Women, and Youth.

## Conflict of Interest

The authors declare that the research was conducted in the absence of any commercial or financial relationships that could be construed as a potential conflict of interest.

## Publisher’s Note

All claims expressed in this article are solely those of the authors and do not necessarily represent those of their affiliated organizations, or those of the publisher, the editors and the reviewers. Any product that may be evaluated in this article, or claim that may be made by its manufacturer, is not guaranteed or endorsed by the publisher.
