# Supplementary material for: How Children and Adolescents Perceive Their Coping With Home Learning in Times of COVID-19: A Mixed Method Approach
Source: Front Psychol. 2021 Nov 30;12:733428. doi: 10.3389/fpsyg.2021.733428 (PMC8669505; doi:10.3389/fpsyg.2021.733428)
Supplement: Supplementary file 1 [file Data_Sheet_1.docx]

Appendix

Table 5: Correlations for children

|  |  | 1. | 2. | 3. | 4. | 5. | 6. | 7. | 8. | 9. | 10. | 11. | 12. | 13. | 14. | 15. | 16. | 17. | 18. |
| --- | --- | --- | --- | --- | --- | --- | --- | --- | --- | --- | --- | --- | --- | --- | --- | --- | --- | --- | --- |
|  | Coping with home learning |  |  |  |  |  |  |  |  |  |  |  |  |  |  |  |  |  |  |
|  | Students’ characteristics |  |  |  |  |  |  |  |  |  |  |  |  |  |  |  |  |  |  |
|  | Age | -.03 |  |  |  |  |  |  |  |  |  |  |  |  |  |  |  |  |  |
|  | Gender | .01 | .04 |  |  |  |  |  |  |  |  |  |  |  |  |  |  |  |  |
|  | Self-efficacy | .15 | .19^*^ | .06 |  |  |  |  |  |  |  |  |  |  |  |  |  |  |  |
|  | Grade | -20* | .09 | .03. | -.36^***^ |  |  |  |  |  |  |  |  |  |  |  |  |  |  |
|  | School joy | .18^*^ | -.14 | 28^**^ | .12 | -.13 |  |  |  |  |  |  |  |  |  |  |  |  |  |
|  | *School context* |  |  |  |  |  |  |  |  |  |  |  |  |  |  |  |  |  |  |
|  | Teacher contact | .07 | -.11 | -.10 | .06 | -.04 | .01 |  |  |  |  |  |  |  |  |  |  |  |  |
|  | amount of time | -.03 | .14 | .03 | -.13 | .06 | -.09 | -.03 |  |  |  |  |  |  |  |  |  |  |  |
|  | School track: primary | -.03 | -.59^***^ | -.10 | -.22^**^ | -.07 | .18^*^ | .08 | -.18^*^ |  |  |  |  |  |  |  |  |  |  |
|  | *Family context* |  |  |  |  |  |  |  |  |  |  |  |  |  |  |  |  |  |  |
|  | HISEI | .14 | .07 | .01 | .13 | -.28^**^ | .17 | -.09 | .11 | -.17^*^ |  |  |  |  |  |  |  |  |  |
|  | Deprivation | -.17^*^ | -.02 | .04 | -.05 | .11 | .04 | .00 | -.02 | .13 | -.39^***^ |  |  |  |  |  |  |  |  |
|  | Migration background | .10 | .04 | .09 | .17 | -.10 | -.02 | -.07 | -.06 | -.08 | .09 | -.24^**^ |  |  |  |  |  |  |  |
|  | Parental support | .14 | -.18^*^ | -.12 | -.06 | .05 | -.07 | .12 | .02 | -.04 | .00 | -.12 | -.12 |  |  |  |  |  |  |
|  | Family climate | .05 | .02 | .02 | .13 | -.25^**^ | .16 | .01 | .08 | -.14 | -.04 | .04 | -.08 | .08 |  |  |  |  |  |
|  | Equipment availability | .35^***^ | .09 | -.08 | -.01 | -.07 | -.03 | -.08 | -.04 | -.19^*^ | .27^**^ | -.35^***^ | .04 | .24^**^ | .03 |  |  |  |  |
|  | Calm place to learn | .29^***^ | .17 | .03 | .06 | .04 | .01 | .03 | -.00 | -.11 | -.02 | -.28^***^ | .05 | .07 | .15 | .34^***^ |  |  |  |
|  | Parental work | -.09 | .00 | .02 | .03 | .18 | .12 | -.04 | .12 | -.02 | -.06 | -.04 | -.06 | .03 | .02 | -.08 | .12 |  |  |
|  | *Peer context* |  |  |  |  |  |  |  |  |  |  |  |  |  |  |  |  |  |  |
|  | Peer support | -.07 | .20^*^ | .17 | .06 | -.12 | -.05 | .12 | -.06 | -.14 | -.09 | -.06 | .07 | .06 | .16 | -.02 | .21^*^ | .04 |  |
|  | Peer group satisfaction | .15 | -.04 | -.09 | .24^**^ | -.34^***^ | -.01 | .13 | -.08 | -.11 | .10 | -.09 | -.05 | .04 | .29^***^ | .21^*^ | .02 | .22 | .02 |

Note. *p < .05, **p < .01, ***p < .001. n = 141, gender (0 = boy; 1 = girl).

Table 6: Correlations for adolescents

|  |  | 1. | 2. | 3. | 4. | 5. | 6. | 7. | 8. | 9. | 10. | 11. | 12. | 13. | 14. | 15. | 16. | 17. |
| --- | --- | --- | --- | --- | --- | --- | --- | --- | --- | --- | --- | --- | --- | --- | --- | --- | --- | --- |
|  | Coping with home learning |  |  |  |  |  |  |  |  |  |  |  |  |  |  |  |  |  |
|  | Students’ characteristics |  |  |  |  |  |  |  |  |  |  |  |  |  |  |  |  |  |
|  | Age | -.08 | 0 |  |  |  |  |  |  |  |  |  |  |  |  |  |  |  |
|  | Gender | .12 | -.03 |  |  |  |  |  |  |  |  |  |  |  |  |  |  |  |
|  | Self-efficacy | .09 | .14^*^ | .01 |  |  |  |  |  |  |  |  |  |  |  |  |  |  |
|  | Grade | -.29^***^ | -.03 | -.13^*^ | -.03 |  |  |  |  |  |  |  |  |  |  |  |  |  |
|  | *School context* |  |  |  |  |  |  |  |  |  |  |  |  |  |  |  |  |  |
|  | Teacher contact | .00 | -.08 | .05 | -.02 | .06 |  |  |  |  |  |  |  |  |  |  |  |  |
|  | Amount of time | .15^*^ | -.10 | .24^***^ | -.01 | -.14^*^ | .07 |  |  |  |  |  |  |  |  |  |  |  |
|  | School track: gymnasium | .06 | .07 | .05 | .06 | -.13^*^ | -.07 | .10 |  |  |  |  |  |  |  |  |  |  |
|  | *Family context* |  |  |  |  |  |  |  |  |  |  |  |  |  |  |  |  |  |
|  | HISEI | .07 | -.10 | -.17^**^ | -.14^*^ | -.18^**^ | -.05 | .05 | .25^***^ |  |  |  |  |  |  |  |  |  |
|  | Deprivation | -.11 | .01 | .04 | -.03 | .20^**^ | .04 | -.08 | -.19^**^ | -.32^***^ |  |  |  |  |  |  |  |  |
|  | Migration background | .04 | .09 | -.05 | .05 | -.20^**^ | -.02 | -.03 | .04 | .11 | -.37^***^ |  |  |  |  |  |  |  |
|  | Parental support | .07 | -.30^***^ | .06 | .00 | -.05 | .10 | .20^***^ | -.10 | .09 | -.03 | -.00 |  |  |  |  |  |  |
|  | Family climate | .29^***^ | -.03 | .02 | .19^**^ | -.11 | -.01 | .21^***^ | .09 | .10 | -.10 | .04 | .30^***^ |  |  |  |  |  |
|  | Equipment availability | .20^**^ | .02 | -.04 | -.01 | -.02 | -.04 | .02 | .09 | .16^*^ | -.22^***^ | .22^**^ | .00 | .05 |  |  |  |  |
|  | Calm place to learn | .34^***^ | -.09 | .01 | .11 | -.09 | .02 | .14^*^ | .13^*^ | .12 | -.10 | .05 | .13^*^ | .35^***^ | .17^**^ |  |  |  |
|  | Parental work | .06 | -.05 | -.15 | .13 | -.03 | .08 | -.12 | -.01 | .03 | .03 | .03 | .06 | -.07 | .11 | -.08 |  |  |
|  | *Peer context* |  |  |  |  |  |  |  |  |  |  |  |  |  |  |  |  |  |
|  | Peer support | .11 | .04 | .07 | .03 | -.02 | -.05 | -.01 | .04 | .12 | .05 | .02 | .10 | .15^*^ | -.01 | .11 | .09 |  |
|  | Peer group satisfaction | .20^**^ | -.03 | .04 | .05 | -.11 | -.08 | .14^*^ | .12^*^ | .19^**^ | -.06 | -.11 | .15^*^ | .28^***^ | .03 | .15^*^ | -.02 | .33^***^ |

Note. *p < .05, **p < .01, ***p < .001.; n=266, gender (0 = boy; 1 = girl).

Table 7: Categories, descriptions, and quotes from parents’ interviews concerning home learning

| Categories | Descriptions | Quotes |
| --- | --- | --- |
| 1. Challenges of home learning 2. finding new arrangements | Parent’s experiences concerning finding new arrangements in balancing work and family life. | “You cannot look after three children and work seven hours at the same time, it does not work like that. [...] Yes, of course, it is a burden, the food has to be cooked, both children have to be taught, the third one also wants his attention and to be looked after. So that is already a higher burden than normal weeks.” (Father of Bernd, Jonas, and Benny, 6y, 9y, and 11y) |
| 1. Challenges of home learning 2. lack of time | Parent’s experiences concerning lack of time to deal with home learning. | “I always get stressed out a little bit, that we're always a little bit behind with the tasks.” (Mother of Heike, 11y) |
|  |  | “I think that if we were to sit next to her, as intensively as with her brother, [...] she would certainly be able to do more [...], but that is simply not possible. [...] We simply had to set priorities so that we could keep Hannes (8y) on track somehow, who has problems at school anyway. And unfortunately that is at the expense of Maria’s school, it has to be said quite clearly. But on the other hand, we have to get our work done.” (Mother Maria, 11y) |
| 1. Children’s ability to learn independently | Parent’s reports concerning their children’s ability to work independently during home learning. | “Because, as I said, with Andrea I don't have to do much, but it's also usually the case that when she's at school, she's very independent and has all her things well under control, and that's why, that's sort of manageable. She's also learned a lot about media and IT, so she's able to print or scan what she needs and send it back herself. So we hardly notice anything.” (Mother of Andrea) |
|  |  | “Yes, he [Jan] does that pretty much independently already.” (Mother of Jan, 14y) |
| 1. Support from school, teacher contact | Parent’s reports concerning the school’s organization of home learning and teacher contact | “Well, we have experienced teachers where you really have the impression that a bunch of worksheets is emailed without any instructions, explanations, help, and also without demanding feedback (…) and there were teachers who simply addressed the children in a text in such a great way, or the religion teacher who once sent a little voice message. So I think it was very dependent on the person. Whether the teachers reached the children remotely or not.” (Mother Jan, 14y) |
|  |  | “In Andrea’s class it works very well, almost from day one they switched to online school, they are well looked after, and accordingly we have to do less… And I see with the elementary school child, where actually many tasks have fallen on us, and both the support, the motivation, the structuring of the day, and all that had to be done on the side, when you work from home. So there the communication with the teachers has worked less well. And with Andrea in the, the sixth grade the teacher really relieved us of a lot. So that we have felt less of a burden.” (Mother of Andrea) |

Table 7: (continued)

| Categories | Descriptions | Quotes |
| --- | --- | --- |
| 1. Given parental support concerning home learning 2. organisation, daily structure | Supporting the children considering the rearrangement of their daily routines and the organisation of home learning | “I had to send the teachers the stuff, I had to prepare that, I had to look at the learning plans, and so on.” (Mother of Lars, 11y) |
|  |  | “There are different problem areas that play a role. For example that the tasks come separately, each teacher sends something himself, now with the oldest [...]. With the middle one, the plan for the whole week came at once, that's easier than if five teachers, each one sends something, from Saturday to Wednesday something flutters in and is supposed to be returned at some point; so there is no structure in it, nothing is well thought out. Of course, it would make sense if the teachers had coordinated it [...], that is, put together a package of what all has to be ready by when.” (Father of Bernd, Jonas, and Benny, 6y, 9y, and 11y) |
| 1. Given parental support concerning home learning 2. motivation, concentration, work discipline | Supporting the children with regard to maintaining motivation, concentration and work discipline | “Well, we have experienced teachers where you really have the impression that a bunch of worksheets is emailed without any instructions, explanations, help, and also without demanding feedback, and you had the impression, well, the child is now working for the thick folder, so to speak, and does not know at all what for, and so I then partly did not succeed at all in motivating her, only with lots of sweets and any promises.” (Mother Jan) |
|  |  | “The first few times [of home learning] I sat with her and did some of the tasks with her or just watched her so that she did not distract herself.” (Mother of Maya) |
|  |  | “And I mean, in the beginning everybody studied in their own room, but in the end we all sat at the dining table, because this 'studying alone', that doesn't work, and then we all sat down together, and I worked, and the children did their things. And then we could at least exchange ideas, the big one could help the little one, and so on.” (Mother of Lars, 11y) |
| 1. Given parental support concerning home learning 2. dealing with digital technology | Supporting the children in dealing with digital technology | “My wife, in particular, structured [incoming tasks] [...] and then printed everything out accordingly and put it down and then sent it back again if necessary.” (Father of Bernd, Jonas and Benny, 6y, 9y, and 11y) |
|  |  | “She has also learned a lot about media and IT, so that she can print or scan what she needs herself and send it back. [...] [B]ut for an elementary school child, [Andrea’s sister (9y)], that means [...] [however] being asked over and over again, ‘What do I do now?’ and why something did not work [...]. And that alongside somehow teleconferencing and email [...] and that really makes you very tired.” (Mother of Andrea, 11y) |
|  |  | “She [younger sister of Jan] has no connection to it at all yet”. (Mother of Jan, 14y) |

Table 7: (continued)

| Categories | Descriptions | Quotes |
| --- | --- | --- |
| 1. Given parental support concerning home learning 2. learning tasks, content support | Supporting the children with regard to their learning tasks | “Yes, so of course we are lucky again that he [Holger, 12] works RELATIVELY independently. So it was always rather like that, that you had to motivate him. Once he was working on it, he was able to work out most of it on his own. But of course, let's say that with some questions, that is, in math, it was a bit difficult, so the arithmetic mean and median and such stories, so we had to support a bit. But otherwise, yes, the effort was kept within limits. [...] I rather felt the burden of having to keep him in a good mood and psychologically stable.” (Mother of Thomas, 14y and Holger, 12y) |

Table 8: Categories, descriptions, and quotes from children interviews concerning home learning

| Categories | Descriptions | Quotes |
| --- | --- | --- |
| 1. Challenges of home learning 2. feelings about the new situation | Children’s reports concerning the challenges and feelings about the new home learning situation. | “Well, I can't see any friends now, of course, and with school and so, a lot has changed, not necessarily in a positive way, of course; and I still found it very uneasy and critical at the beginning, because you didn't know exactly what to do now, but over time it all came together.” (Benny, 11y) |
|  |  | “We get the assignments from school by email, and you have a bit of a feeling that you somehow get more assignments than you would normally if you went to school, and you also cannot learn or understand things as well as in normal lessons.” (Maja, 11y) |
| 1. Challenges of home learning 2. motivation, concentration | Children’s reports concerning motivation and concentration during home learning | “When I am home alone, […] it is difficult to concentrate [...]. It’s the same at school, but at home, I have something like a mobile phone or TV, where I would rather do that and think it does not matter so much if I miss one day, but there [at home] I somehow miss three days.” (Maria, 11y) |
| 1. Children’s experiences of learning success | Children’s reports concerning their learning success | “We managed everything that was assigned, even additional tasks.” (Marcus, 10y) |
|  |  | “Yes, and I think I also learned a little bit more at home because I could ask more questions there.” (Heike, 11y) |
| 1. Support from school, teacher contact | Children’s reports concerning the school’s organization of home learning and teacher contact | “I would have actually wished for a bit more [video conferences], because I always thought it was such a change, not just sitting at a desk, because I somehow could not motivate myself so well to just do homework alone at a desk, so I thought that was pretty cool.” (Jan, 14y) |
| 1. Received parental support concerning home learning 2. organisation, daily structure | Children’s reports concerning parental support received in the rearrangement of their daily routines and the organisation of home learning | “How am I supposed to manage all the tasks? That was a big problem at the beginning; but then I and my mother also found appropriate solutions with apps that show the time, that I worked for three hours and 30 minutes every day, and then we also made plans, and then also with such a list to check off, which then worked very well with school after a while” (Benny, 11y) |
| 1. Received parental support concerning home learning 2. motivation, concentration, work discipline | Children’s reports concerning parental support received to maintain motivation, concentration and work discipline | “And that's when I need help, when I somehow, somehow don't have my head in the right place (laughs) and I feel like I've forgotten everything! And then I also ask my parents, and they can usually help me.“ (Maja, 11y) |
| 1. Received parental support concerning home learning 2. dealing with digital technology | Children’s reports concerning parental support received in dealing with digital technology | “So we always got weekly plans, which they sent by e-mail, and which we then printed out sometimes; and we also sometimes skyped, so mostly on Wednesday or on Thursday, we then had math via Skype. And yes, sometimes we also got worksheets sent to us, which we were then supposed to print out.” (Heike, 11y) |

Table 8: (continued)

| Categories | Descriptions | Quotes |
| --- | --- | --- |
| 1. Received parental support concerning home learning 2. learning tasks, content support | Children’s reports concerning parental support received with regard to their learning tasks | “That you can always see your friends, that you can also talk to the teachers if you don't understand an assignment, for example, then I probably ask, then I always ask my brother or my mother most of the time.” (Lars, 11y) |
| 1. Connectedness and support from peers    1. using media to connect | Children’s reports concerning the connection with peers via media | “Yes, we have such a program, so ‘Discord’ is the name of it, which we also always use when we play computer games or something (laughs), and then we always met at a time and just did the tasks together.” (Jan, 14y) |
| 1. Connectedness and support from peers 2. motivation, concentration, work discipline | Support from peers with regard to maintaining motivation, concentration and work discipline | “We talked a lot on the phone [...] and then just did the tasks together when studying because it’s just a bit stupid alone.” (Maja, 11y) |
| 1. Connectedness and support from peers 2. learning tasks | Support from peers with regard to learning tasks | “So, for example, if my friend did not know something or I did not know something, then we gave each other tips or something about what it could be, and then we sometimes, so often, came up with the right solution.” (Marcus, 11y) |
|  |  | “We also wrote a lot like that, if tasks were not clear so that I and my friend did not know and his mother did not know either, we sometimes asked the smartest from our class.” (Marcus, 11y) |
